# Triggering Apoptotic Death of Human Epidermal Keratinocytes by Malic Acid: Involvement of Endoplasmic Reticulum Stress- and Mitochondria-Dependent Signaling Pathways

**DOI:** 10.3390/toxins7010081

**Published:** 2015-01-09

**Authors:** Yu-Ping Hsiao, Wan-Wen Lai, Shi-Bei Wu, Chung-Hung Tsai, Sheau-Chung Tang, Jing-Gung Chung, Jen-Hung Yang

**Affiliations:** 1 Institute of Medicine, Chung Shan Medical University, 402 Taichung, Taiwan; E-Mails: missyuping@gmail.com (Y.-P.H.); hauhauma@yahoo.com.tw (W.-W.L.); pathology@csmu.edu.tw (C.-H.T.); 2 Department of Dermatology, Chung Shan Medical University Hospital, 402 Taichung, Taiwan; 3 Department of Biochemistry and Molecular Biology, National Yang-Ming University, 112 Taipei, Taiwan; E-Mail: labboy700110@gmail.com; 4 Department of Pathology, Chung Shan Medical University Hospital, 402 Taichung, Taiwan; 5 Department of Dermatology, Buddhist Tzu Chi General Hospital, 907 Hualien, Taiwan; E-Mail: s6160051@yahoo.com.tw; 6 School of Medicine, Tzu Chi University, 907 Hualien, Taiwan; 7 School of Biological Science and Biotechnology, China Medical University, 404 Taichung, Taiwan; E-Mail: jgchung@mail.cmu.edu.tw

**Keywords:** malic acid (MA), HaCaT cells, seahorse XF 24 analyzer, apoptosis

## Abstract

Malic acid (MA) has been commonly used in cosmetic products, but the safety reports in skin are sparse. To investigate the biological effects of MA in human skin keratinocytes, we investigated the potential cytotoxicity and apoptotic effects of MA in human keratinocyte cell lines (HaCaT). The data showed that MA induced apoptosis based on the observations of DAPI staining, DNA fragmentation, and sub-G1 phase in HaCaT cells and normal human epidermal keratinocytes (NHEKs). Flow cytometric assays also showed that MA increased the production of mitochondrial superoxide (mito-SOX) but decreased the mitochondrial membrane potential. Analysis of bioenergetics function with the XF 24 analyzer Seahorse extracellular flux analyzer demonstrated that oxygen consumption rate (OCR) was significantly decreased whereas extracellular acidification rate (ECAR) was increased in MA-treated keratinocytes. The occurrence of apoptosis was proved by the increased expressions of FasL, Fas, Bax, Bid, caspases-3, -8, -9, cytochrome *c*, and the declined expressions of Bcl-2, PARP. MA also induced endoplasmic reticulum stress associated protein expression such as GRP78, GADD153, and ATF6α. We demonstrated that MA had anti-proliferative effect in HaCaT cell through the inhibition of cell cycle progression at G0/G1, and the induction of programmed cell death through endoplasmic reticulum stress- and mitochondria-dependent pathways.

## 1. Introduction

Malic acid (MA), a sort of alpha-hydroxy acids (AHAs) found in fruits and many vegetables, has been commonly used in cosmetics and chemical peeling agents [1,2,3,4]. Malic acid is used for light-damaged or dry skin, and acne [3]. Over 50 cosmetic formulations across a range of products have contained MA [4]. However, safety concerns of the adverse reactions of AHAs including redness, swelling, burning, pruritus, phototoxicity, and facial hyperkeratosis were pronounced [2,5,6,7,8]. Malic acid may induce skin and ocular irritation [4] and the biological and molecular effects of malic acid in human keratinocytes are still uncertain. In this study, we investigated the potential cytotoxicity and apoptotic effects of malic acid in human keratinocyte cell lines (HaCaT) and normal human epidermal keratinocytes (NHEKs).

## 2. Results

### 2.1. Anti-Proliferative Effects of MA on Cell Morphology and Viability of HaCaT Cells

**Figure 1:**
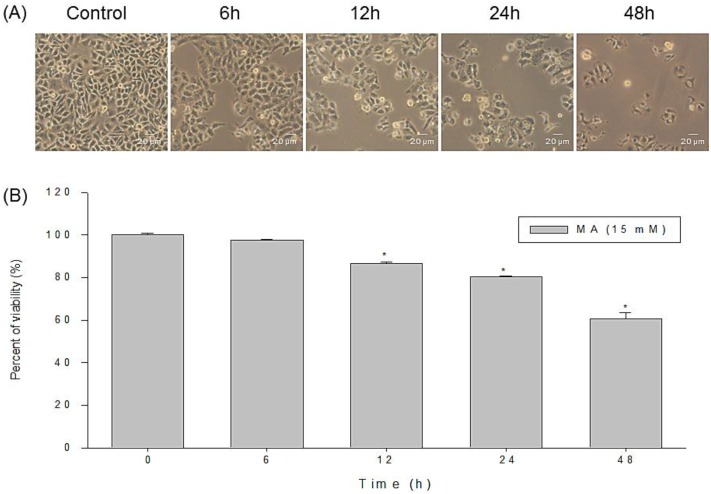
Malic acid induced cells’ morphological changes and decreased the number of total viable human keratinocytes (HaCaT cells). Cells were incubated with or without 15 mM of malic acid for 6, 12, 24, and 48 h, and then were examined and photographed by a phase-contrast microscope (**A**) and were harvested for determination the percentage of viable cells by flow cytometry (**B**). Data are presented as means ± S.D. of the results from three independent experiments (* *p* < 0.05* vs.* the indicated group).

HaCaT cells were treated with MA at various concentrations (0, 10, 12.5, 15, 17.5, and 20 mM) for 24 h and 48 h, and at 15 mM for different incubation periods (0, 6, 12, 24, and 48 h). The cells were photographed and were collected before being stained by PI and were analyzed for viability by flow cytometry. A larger proportion of cells were swelling and round-out indicating necrosis, and some revealed shrinkage of cells with unclear nuclei suggesting apoptosis (Figure 1A). The effect of MA on viability of HaCaT cells revealed a time-dependent (Figure 1B) and dose-dependent manner (data not shown). The half maximal inhibitory concentration (IC50) value of MA was close to 15 mM at a 24-h exposure. Thus, this concentration at 15 mM of MA was applied for all subsequent experiments.

### 2.2. MA Induced Cell Cycle Arrest and Apoptosis in HaCaT Cells and NHEKs

**Figure 2:**
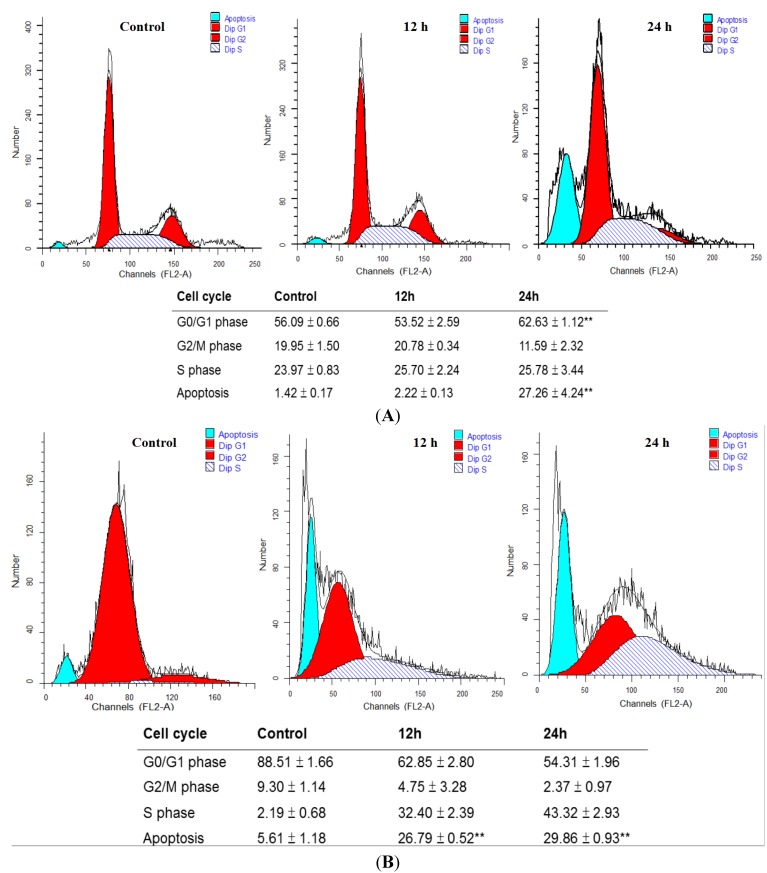
Malic acid changed the DNA content and induced apoptosis in HaCaT cells (**A**); and normal human epidermal keratinocytes (NHEKs) (**B**). Cells were treated with 15 mM of MA for 0, 12, and 24 h. The cell cycle distribution and sub-G_1_ group (apoptosis phase) were determined using flow cytometric analysis and obtained from three independent experiments with similar results. Data are presented as means ± S.D. of the results from three independent experiments (** *p* < 0.01* vs.* the indicated group).

The flow cytometry disclosed that treatment of MA at 15 mM increased the proportion of cells at G0/G1 phases after 24 h in HaCaT cells (Figure 2A). MA also induced a distinct subG1 peak, which represents the population of apoptotic cells in HaCaT cells and normal human epidermal keratinocytes (NHEKs) (Figure 2B).

### 2.3. MA Induced Cell Damage Examined by DAPI Staining and DNA Fragmentation

**Figure 3:**
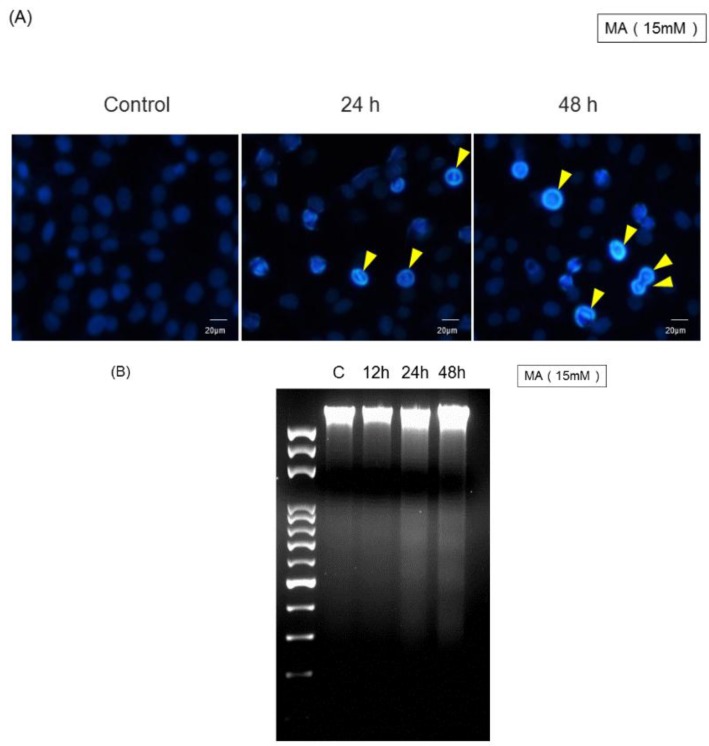
Effects of malic acid on DNA damage of HaCaT cells. Cells were treated with 15 mM of malic acid for various time periods. Cells were harvested individually and then were measured with DAPI staining (**A**); and DNA gel electrophoresis (**B**) as described in Materials and Methods. Data are presented from three independent experiments.

Apoptotic cells demonstrated with DAPI staining were higher in intensity than that in non-apoptotic live cells. There was an increase in the number of fragmented nuclei in MA-treated HaCaT cells at 24 h and 48 h (Figure 3A). MA-induced cell apoptosis was confirmed using a DNA fragmentation assay. HaCaT cells treated with 15 mM of MA for 24 and 48 h showed a significant increase of DNA fragmentation compared to the controls (Figure 3B).

### 2.4. Intracellular Superoxide Dynamics of HaCaT Cells Incubated in Malic Acid

**Figure 4:**
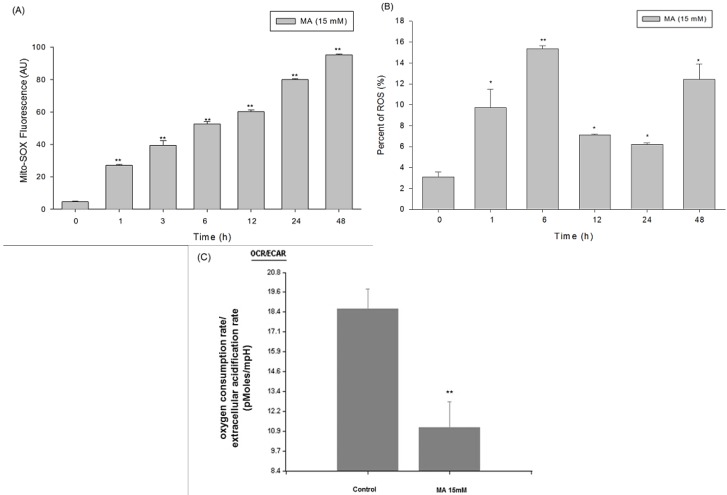
Increase of oxidative stress and anaerobic glycolysis in HaCaT cells treated with malic acid. (**A**) Malic acid increased mitochondrial ROS production in HaCaT cells detected by flow cytometry; (**B**) malic acid increased ROS production in terms of increasing 2',7'-dichlorodihydrofluorescein (DCF) fluorescence intensity in HaCaT cells detected by flow cytometry; (**C**) The mean values of oxygen consumption rate (OCR) and extracellular acidification rate (ECAR) were measured in real-time by a Seahorse XF24 Analyzer. Data are presented as means ± S.D. of the results from three independent experiments (** *p* < 0.01* vs.* the indicated group, *).

HaCaT cells were incubated in malic acid dispersions and incubated with MitoSOX™ Red—a novel fluorescent indicator for the selective measurement of mitochondrial superoxide (O2-·) production in cells (Invitrogen, Carlsbad, CA, USA). So, the effect of culturing HaCaT cells in malic acid (15 mM) medium for different time periods on mitochondrial superoxide (O2-·) was assessed using MitoSox. Under basal conditions, malic acid-cultured cells produced a significantly greater amount of mitochondrial superoxide (O2·-) than those of control group in a time-dependent manner (Figure 4A). MA increased production of ROS in terms of increasing 2',7'–dichlorodihydrofluorescein (DCF) fluorescence intensity in HaCaT cells (Figure 4B).

### 2.5. Measurement of Oxygen Consumption Rate (OCR) and Extracellular Acidification Rate (ECAR) of Malic Acid-Treated HaCaT Cells by Seahorse XF24 Analyzer

To assess whether the malic acid had an effect on basal oxygen consumption and extracellular acidification in human skin, Seahorse XF24 Analyzer were used. There was no effect on basal oxygen consumption rate (OCR) and extracellular acidification rate (ECAR) of malic acid-treated HaCaT cells as compared to those of control without malic acid treatment. In addition, malic acid-treated HaCaT cells had higher mitochondrial stress in response to oligomycin and DNP (*p* > 0.05). Malic acid-treated HaCaT cells had lower OCR (more respiration) and greater glycolysis (ECAR) than control group (*p* < 0.05), suggesting greater glucose oxidation and glucose utilization in malic acid-treated HaCaT cells (Figure 4C).

### 2.6. Effects of MA on Caspase-3 and Inhibition of MA-Induced Apoptosis by the Caspase-3 Inhibitor Z-DEVD-FMK in HaCaT Cells

**Figure 5:**
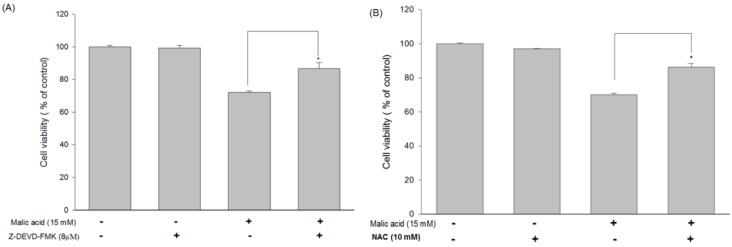
Malic acid stimulated ROS, caspase-3, -8 and -9 activities and altered viability after pre-incubation with specific inhibitors in HaCaT cells. (**A**) Cells were incubated with 15 mM malic acid for 24 h before exposure in presence and absence of the specific inhibitors of caspase-3 (Z-DEVD-FMK) for 3 h to measure the viability in HaCaT cells; (**B**) Malic acid-treated HaCaT cells were in the presence and absence of N-acetyl-L-cysteine (NAC) at a concentration of 10 mM. Cell viabilities were then determined as described above. Data are presented as means ± S.D. of the results from three independent experiments (* *p* < 0.05* vs.* the indicated group).

MA affects the activities of caspase-8, -9 and -3 in HaCaT detected by cytometry (Figure S1). To confirm whether MA induced apoptosis through the activation of caspase-3 in HaCat cells, cells were pretreated with or without the caspase-3 inhibitor (Z-DEVD-FMK) and then were treated with 15 mM of MA and were harvested and assessed by flow cytometric assay and the results are shown in Figure 5A. These results showed that MA induced apoptosis via caspase-3 dependent pathway. Besides, This ROS was completely reduced in the presence of *N*-acetyl-l-cysteine (NAC) as shown in Figure 5B.

**Figure 6:**
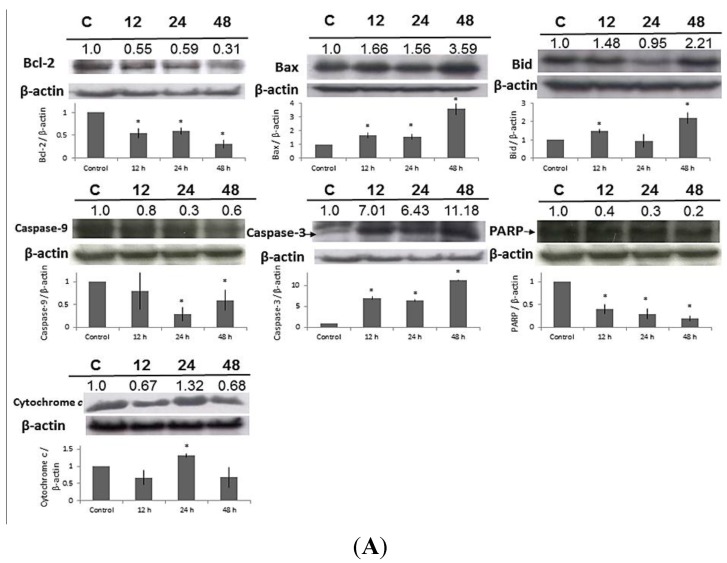

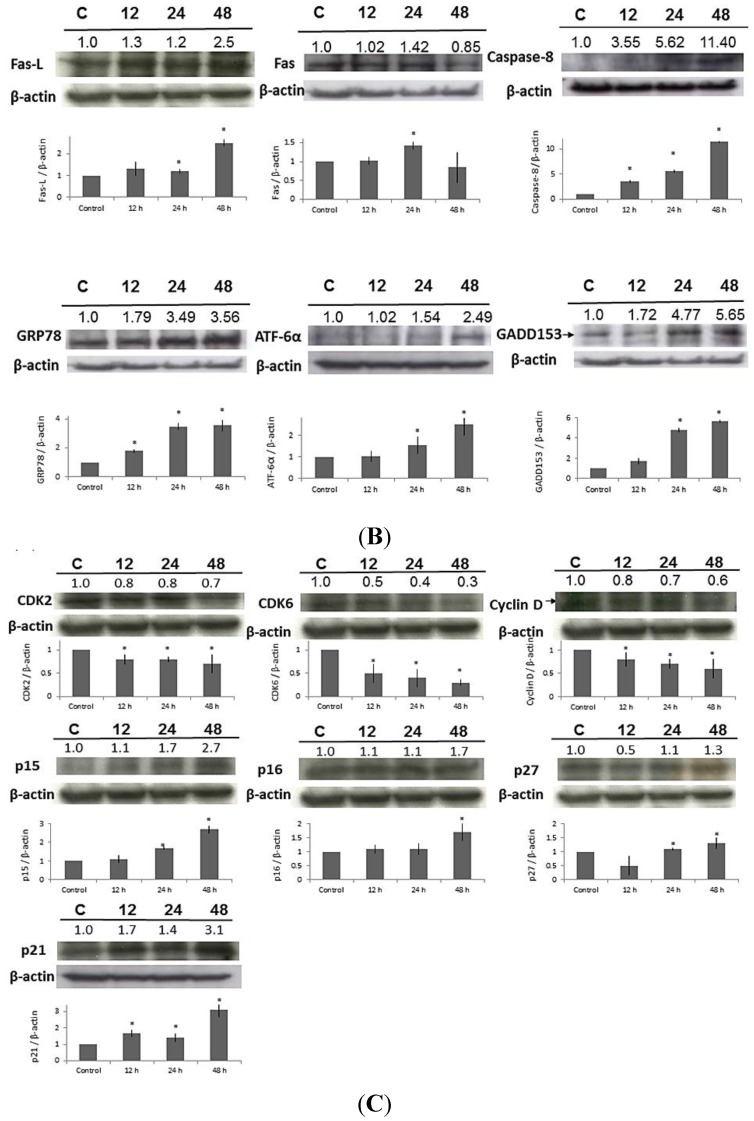
Western blot demonstrated the increase of Fas, FasL, Bax, Bid, caspase-3, -8, -9, cytochrome *c*, GADD153, GRP78, ATF6α, and decrease of Bcl-2 in HaCaT cells exposed to malic acid at 15 mM for different incubation periods. Data are presented as means ± S.D. of the results from three independent experiments (* *p* < 0.05* vs.* the control group).

### 2.7. Effect of MA on the Expressions of Apoptosis-Related Proteins in HaCaT Cells

HaCaT cells were treated with MA at 15 mM for different time periods (0, 12, 24, and 48 h). MA inhibited the levels of Bcl-2, PARP, but promoted the expressions of Bax, Bid, caspase-3, cytochrome c (Figure 6A). MA increased the expression of Fas-l, Fas, and caspase-8 and (Figure 6B). These findings show that MA induces apoptosis via the caspase- and mitochondrial-dependent pathway in HaCaT cells. Furthermore, MA promoted the expression of GRP78, ATF-6α, and GADD153 (Figure 6B), indicating that MA induced apoptosis through ER stress.

### 2.8. Effect of MA on the Expressions of Cell Cycle-Related Proteins in HaCaT Cells

Western blot demonstrated the decrease of CDK 2, CDK 6, and Cyclin D and the increase of P15, P16, P21, and P27; which indicated that MA induced cell cycle arrest at G0/G1 in MA-treated cells at 15 mM for different periods (0, 12, 24, and 48 h) (Figure 6C). 

## 3. Discussion

Topical treatment of photo-aging skin with alpha-hydroxy acids (AHA) has been reported to improve wrinkling, roughness, hyperpigmentation, acne, and rosacea within months of daily application [9,10,11,12]. However, AHAs may induce photosensitivity, increase the number of sunburn cells, and decrease the minimal erythema dose [8,13,14,15]. Although the safety concerns of AHAs were announced by the Ministry of Health and Welfare, R.O.C, chemical peeling agents contained higher concentrations of AHAs (20%–70%) and low pH levels were used in the hospitals and local practitioner’s clinics [16,17]. We have reported that glycolic acid induced programmed cell death in HaCaT cells and also human leukemia cell line HL-60 [8,18]. Although several reports have announced the adverse effects of AHAs, the exact molecular mechanisms of malic acid (MA) in human epidermal keratinocytes still remain unclear, and herein, we investigated MA affecting human epidermal keratinocytes (HaCaT cells).

In the present study, we firstly reported that MA exhibited cytotoxic effects towards HaCaT cells based on the observations of morphological changes (Figure 1A), and decreased the percentage of viable cells (Figure 1B), sub-G1 phase (apoptosis) (Figure 2) and DNA damage (Figure 3). Cells undergoing apoptosis and nuclear fragmentation were also observed in MA-treated cells and these effects are time-dependent.

Apoptosis (programmed cell death) belongs to the parts of DNA damage response [19]. The cellular reaction to genomic instability includes a network of signal transduction pathways referred to as the DNA damage response. Individual damage would trigger DNA damage response through distinct yet overlapping pathways to regulate the cell cycle, DNA repair, transcription, cell cycle progression, and apoptosis [19]. Apoptosis is regulated via the extrinsic pathway (death receptor-dependent pathway) and intrinsic pathway (mitochondria-dependent pathway) [20]. MA treatment promoted caspase-3 activity; however, co-administration of MA and caspase-3 inhibitor, Z-DEVD-FMK, markedly increased cell viability at 24 h in HaCaT cells (Figure 5A). The activation of caspase-3 is often considered as the point-of-no-return in the apoptotic signaling cascades [21].

The results from Western blotting (Figure 6A) indicated that MA increased endoplasmic reticulum stress associated protein expression such as GRP78, GADD153, and ATF6α (Figure 6B). Based on the results of Western blotting (Figure 6A) and flow cytometry (Figure S1), MA also promoted the expression of Fas and FasL and active form of caspase-8; MA inhibited the protein level of Bcl-2 but promoted the Bax, and both proteins led to the release of cytochrome *c* from mitochondria and activation of caspase-9 and -3, PARP cleavage, resulting in the apoptotic death which is mediated through the mitochondrial pathway. MitoSOX™ Red is a cell permeable dye that forms a highly fluorescent product upon oxidation [22,23]. Owing to its lipophilic triphenyl phosphonium cation, MitoSOX™ Red (Invitrogen, Carlsbad, CA, USA) is selectively targeted to mitochondria—the major source of ROS in cells—where it can be oxidized by superoxide before exhibiting red fluorescence upon binding to nucleic acids [22,23]. The XF 24 analyzer Seahorse is a novel extracellular flux analyzer used to analyze mitochondrial bioenergetics [24,25,26]. Mitochondrial oxygen consumption rate (OCR) is used to measure the oxidative phosphorylation and extracellular acidification rate (ECAR) as a measure of Glycolysis [24]. MA modulated mitochondrial biogenesis based on the fact that MA increased mitochondrial ROS MitoSOX™ Red (Invitrogen, Carlsbad, CA, USA) and decreased oxygen consumption rate/extracellular acidification rate in HaCaT (Figure 5).

In this study, the concentration of MA was 15 mM (0.18%) and is remarkably lower than the concentration (<1%) in cosmetics. Although the skin tissue levels of MA from the topical application of MA are lacking, our data clearly demonstrated that MA had anti-proliferative and apoptotic effects in human keratinocytes* in vitro*. Therefore, we are concerned about the potential adverse effects for long-term use of MA-containing resurfacing or peeling products. The limitation of our study is that HaCaT cells have intrinsic differences in comparison to those in primary normal human keratinocytes. The phases of cell cycle arrest were not similar in HaCaT cells and NHEKs (Figure 2B). Rate of cell proliferation in NHEKs was much slower than in HaCaT cells [27], and the percentage of G1-arrested cells was prominent in HaCaT cells than in NHEKs under oxidative stress [28]. That may explain part of reason why MA induced G0/G1 phases arrest in HaCaT cells, but caused S phase arrest in NHEKs. Nevertheless, NHEKs have a limited life span and could only survive for several passages. HaCaT is a convenient substitute and can maintain a non-tumorigenic phenotype [29]. HaCaT cells are excellent models to study the regulation of keratinocyte physiology by the circadian clock* in vitro* system [30].

In summary, we demonstrated that the effects of MA in HaCaT cells were as follows: (1) To cause cytotoxicity and morphological changes in immortalized human keratinocytes, and the inhibitory effect of MA in HaCaT viability was dose- and time-dependent; (2) To induce the cell cycle arrest at the G0/G1 transition via Cyclin D, CDK6, CDK2, P15, P16, P21, and P27 checkpoints; (3) To induce apoptosis based on the result of flow cytometry, DAPI staining, DNA gel electrophoresis, the induction of apoptosis was proved by the declined expressions of Bcl-2 and PARP and the increased expressions of ROS, Bax, Bid, caspases-3, -8, GRP78, GADD153, and ATF6α.

## 4. Materials and Methods

### 4.1. Chemicals and Reagents

Malic acid was obtained from Sigma Chemical (St. Louis, MO, USA). Dimethyl sulfoxide (DMSO), potassium phosphate, and TE buffer (10 mM Tris-HCl, pH 8, 1 mM EDTA) were purchased from Merck Co. (Darmstadt, Germany). Trypan blue, Tris-HCl, triton X-100, propidium iodide (PI) and ribonuclease A were obtained from Sigma Chemical Co. (St. Louis, MO, USA). Fetal bovine serum, penicillin-streptomycin, trypsin-EDTA and glutamine were obtained from Gibco BRL (Grand Island, NY, USA). Caspase-3 activity assay kit was purchased from Roche Diagnostics (Mannheim, Germany). All of the chemicals used were reagent grade.

### 4.2. Human Immortalized Keratinocytes (HaCaT) Cell Line and Normal Human Keratinocytes (NHEKs)

HaCaT cells (Cell Lines Service, Eppelheim, Germany) were cultured on Dulbecco’s modified Eagle’s medium (DMEM) supplemented with 1% L-glutamine, 25 mM HEPES, 10% fetal bovine serum and 1% penicillin-streptomycin (Gibco, Carlsbad, CA, USA) at 37 °C in a humidified incubator with 5% CO_2_ atmosphere [31]. Normal human epidermal keratinocytes (NHEKs) were obtained from Cell Applications, Inc. San Diego, USA and were cultured in Keratinocyte-SFM (Gibco BRL/Invitrogen, Carlsbad, CA, USA) supplemented with recombinant epidermal growth factor (0.1–0.2 ng/mL), bovine pituitary extract (20–30 mg/mL), and 1% penicillin/streptomycin in a humidified atmosphere at 37 °C and 5% CO2. The second- to fourth-passage cells were used in the experiments.

### 4.3. Morphological Changes and Viability of HaCaT Cells Treated with or without MA

We observed the cell morphology under a phase-contrast microscope, and collected the HaCaT cells stained with PI and detected the viability with a flow cytometer (Becton-Dickinson, San Jose, CA, USA) equipped with an argon laser at 488 nm wavelength. We calculated the percentages of cell viability by using CellQuest software (Becton-Dickinson) and flow cytometer (Becton-Dickinson). Approximately 10 μL of cell suspensions in PBS were mixed with 40 μL of propidium iodine (PI), and the numbers of stained (dead cells) and unstained cells (live cells) were counted using flow cytometric assay. We used CellQuest to calculate the ratios of viable cells (PI negatively stained)/total cells as the percentages of cell viability. Data represent mean ± SD of the results from three experiments (*n* = 3).

### 4.4. Flow Cytometric Analysis of Cell Cycle and Apoptosis of HaCaT Cells and NHEKs Treated with Different Concentrations of MA

HaCaT cells and normal human epidermal keratinocytes (NHEKs) were treated with MA (15 mM) in an incubator for different 0, 12, and 24 h before the cells were harvested by centrifugation. The cells were fixed gently by 70% ethanol at 4 °C for overnight and were then resuspended in PBS containing 40 mg/mL PI and 0.1 mg/mL RNase and 0.1% Triton X-100 in a dark room. After incubation at 37 °C for 30 min, the cell cycles and apoptosis were analyzed with a flow cytometer.

### 4.5. Examination of Apoptosis by 4,6-Diamidino-2-Phenylindole Dihydrochloride (DAPI) Staining and Determination of DNA Fragmentation by Gel Electrophoresis

Approximately 2 × 10^5^ cells/well of HaCaT cells had been treated with MA at 15 mM for 0, 24, and 48 h. Cells in each wells were stained with DAPI (4,6-diamidino-2-phenylindole dihydrochloride) before fixation with 3.7% formaldehyde. The cells were then washed with PBS and examined by fluorescence microscopy (Nikon Coolpix 4500, Tokyo, Japan, 200×).

Total DNA was isolated from each sample using a DNA purification kit (Genemark Technology, Tainan, Taiwan) and resolved in an 1.8% agarose gel containing 0.3 mg/mL ethidium bromide (Sigma-Aldrich, St. Louis, MO, USA) in a 0.5 X TBE buffer (0.045 mol/L Tris, 0.045 mol/L boric acid, 1 mmol/L Na_2_ EDTA acid, pH 8.3) after electrophoresis for 45 min. The DNA bands were visualized, examined, and photographed as described previously.

### 4.6. Determination of Mitochondrial ROS Production and Intracellular ROS by Flow Cytometry

Cells were rinsed at least three times with sterile PBS and loaded with a solution of MitoSOX™ Red (Molecular Probes, Invitrogen, Carlsbad, CA, USA). Specifically, cells were incubated for 10 min at 37 °C in a 5 μM MitoSOX™ Red (Invitrogen, Carlsbad, CA, USA) solution. Next, cells were rinsed three times with PBS, harvested with 500 μL of trypsin-EDTA solution, centrifuged at 5000 rpm for 5 min, and resuspended in 3 mL of fresh 2% (*v*/*v*) FBS/PBS. Finally, cell suspensions were filtered through a 30-μm PreSeparation filter (Miltenyi Biotec, Bergisch Gladbach, Germany). Fluorescence-based flow cytometry was performed using a Becton Dickinson FACSCalibur^®^ (BD Biosciences, Totowa, NJ, USA) flow cytometer equipped with a 488 nm laser. MitoSOX™ Red (Invitrogen, Carlsbad, CA, USA) fluorescence (λMax = 590 nm) was detected over the range of 564–606 nm and the background fluorescence was detected over the range of 515–545 nm. Besides, the levels of intracellular ROS of the HaCaT cells were determined by flow cytometry. HaCaT cells were treated with or without MA (15 mM) for different time periods (0, 1, 6, 12, 24, and 48 h). The cells were harvested and washed twice, resuspended in 10 µM 2,7-dichlorodihydrofluorescein diacetate (Sigma-Aldrich, St. Louis, MO, USA) and incubated at 37 °C for 30 min and the levels of ROS were analyzed by flow cytometry. Intracellular ROS was detected by means of an oxidation-sensitive fluorescent probe (DCFH-DA). In the presence of ROS, DCFH-DA was subsequently transferred to DCF and emitted a green fluorescent signal detected by flow cytometry. All quantitations were performed using CellQuest 7.5.3 software (BD Biosciences, Totowa, NJ, USA); in each experiment, well over 10,000 cells were analyzed.

### 4.7. Measurement of Bioenergetic Parameters of Mitochondria by Seahorse XF24 Analyzer

An XF24 Analyzer (Seahorse Bioscience, North Billerica, MA, USA) was used to measure the bioenergetic function of the primary culture of skin fibroblasts [25]. The XF24 Analyzer can create a transient 7-μL chamber to the cells cultured in a 24-well microplate, and the oxygen consumption rate (OCR) and extracellular acidification rate (ECAR) were monitored real-time in an incubation chamber at 37 °C. Briefly, a seeding density of 16,000 skin cells per well was chosen and the culture medium was replaced 1 h prior to measurement by the assay medium that contained un-buffered DMEM (pH 7.4). The program of Seahorse XF24 Analyzer was set according to the manufacturer’s recommendation and the data are expressed in pmol/min/10^4^ cells for OCR and in mpH/min/10^4^ cells for ECAR, respectively, to allow comparison between independent experiments.

### 4.8. Determination the Effect of Caspase-3 Inhibitor Z-DEVD-FMK and ROS Inhibitor NAC on Cell Viability in MA-Treated HaCaT Cells

HaCaT cells were treated with MA (15 mM) for 24 h, and then with lysis buffer (1% Triton X-100, 0.32 M sucrose, 5 mM EDTA, 10 mM Tris-HCl, pH 8, 2 mM dithiothreitol, 1 mM phenylmethylsulfonyl fluoride, 1 µg/mL aprotinin, and 1 mg/mL leupeptin) for 30 min at 4 °C followed by centrifugation at 10,000× *g* for 30 min. To examine whether or not caspase-3 activation was involved in cell viability triggered by MA, HaCaT cells had been pretreated with Z-DEVD-FMK (caspase-3 inhibitor) 3 h prior to treatment with MA. MA-treated HaCaT cells were in the presence and absence of *N*-acetyl-l-cysteine (NAC) at a concentration of 10 mM. Cell viabilities were then determined as described above [7].

### 4.9. Western Blotting for Examining the effect of MA on Expressions of Apoptosis-Related and Cell Cycle Checkpoints-Related Proteins

Total proteins were collected from HaCaT cells treated with MA for different time period (0, 12, 24, and 48 h). Western blotting was used to examine the expression levels of the apoptosis-related proteins including Bax, Bcl-2, Bid, PARP, caspases-3, caspase-4, caspases-8, caspase-9, cytochrome *c*, Fas, Fas-l, ATF-6α, GADD153, GRP 78, and cell cycle-related proteins including CDK 2, CDK 6, cyclin d, P15, P16, P21, P27 by sodium dodecylsulfate polyacrylamide gel electrophoresis (SDS-PAGE). The protein bands were visualized and measured by using Alpha Image 1220 Documentation and Analysis System (Alpha Innotech, San Leandro, CA, USA).

### 4.10. Statistical Analysis

All experiments were performed in triplicate and presented as means ± SD. Statistical analyses were performed using one-way analysis of variance (ANOVA, SAS software, SAS Institute Inc., Cary, NC, USA, version 9.2). followed by Tukey post hoc test to determine significant differences among the groups. The difference was considered significant when *p* < 0.05.

## 5. Conclusions

**Figure 7:**
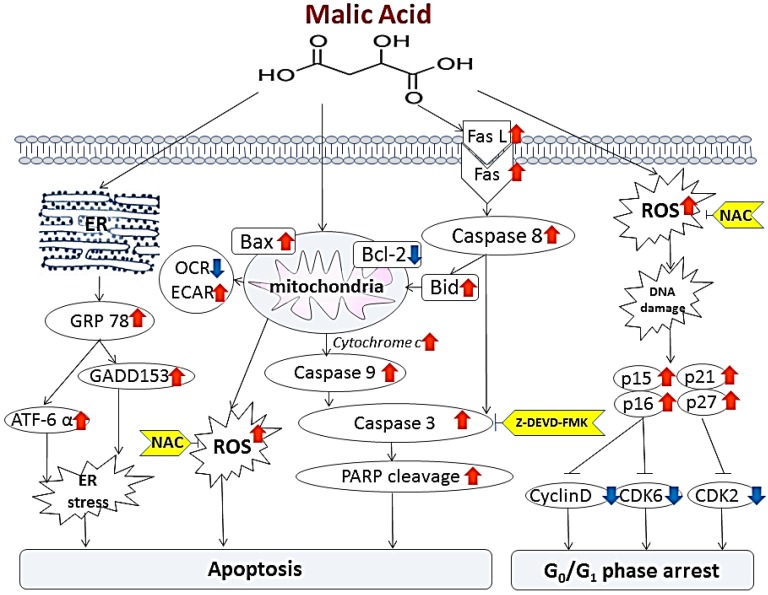
The proposed signaling pathways of malic acid-induced G0/G1 phase arrest and apoptosis in human epidermal keratinocytes HaCaT cells.

In conclusion, we demonstrated that MA induced cytotoxic effects and anti-proliferative effect in HaCaT cell through the inhibition of cell cycle progression and the induction of programmed cell death* in vitro*. We also demonstrated that MA-induced apoptosis were through multiple molecular pathways including involvement of endoplasmic reticulum stress- and mitochondria-dependent signaling pathways (Figure 7).

## Supplementary Materials

Supplementary materials can be accessed at: http://www.mdpi.com/2072-6651/7/1/0081/s1.
